# The First Word

**DOI:** 10.4103/0972-124X.55835

**Published:** 2009

**Authors:** D. Arunachalam

**Affiliations:** *Editor, Journal of Indian Society of Periodontology, H 11 A, South Avenue, Thiruvanmiyur, Chennai - 600041, India. E-mail: smilesindia@gmail.com*


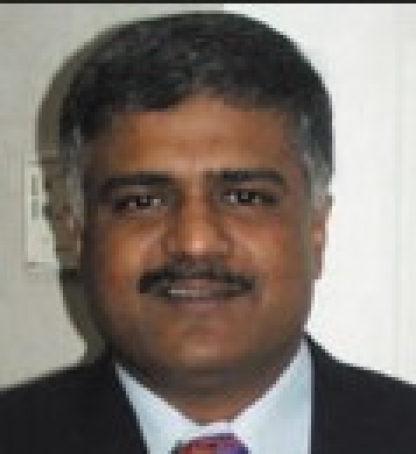


Plus ça change, plus c'est la même chose” or “the more things change, the more they stay the same” Jean-Baptiste Alphonse Karr (Les Guêpes, January 1849).

I have often wondered if this is not true in our field of Periodontics too. Recently, I was pleasantly surprised to receive a phone call from Prof. A Jayakumar, a guide to many of us and a great and warm friend to all around him. Our conversation, after pleasantries, drifted on to Periodontics as usual, and then came the clincher — are we doing anything differently in Periodontics than say, 30 years ago? Both of us converged on the fact that if we were, it was precious little. I have requested Dr. Jayakumar to let our readers have the benefit of a Guest Editorial by him, covering the last 30 years of Periodontics in India, as seen through his eyes. Hope he will oblige and we will have the benefit of his immense wisdom.

However, one thing is certain; other disciplines in dentistry have now started viewing Periodontics more seriously. I believe this is largely because of the utterly impotent feeling that arises out of not being able to complete restorative, prosthodontic, or implant care with a sense of complete success, without having periodontology to round it off. Definitely, there is considerable scope for the sharing of expertise from various fields, to establish higher therapeutic goals and attain better outcomes. The key to this probably lies in an interdisciplinary model of periodontal therapy and care in which specialists of many fields cooperate. However, the involvement of many specialists without proper coordination and agreement in the therapeutic goals, could spell disaster. The key to the interdisciplinary model is to form a correct team of individuals.

The current issue has several articles where interdisciplinary Periodontics and a team approach seem to be the dominant theme. Forming an interdisciplinary team seems to be the solution to understanding the possibilities available in treatment planning for patients with complex problems. Having such a team to call upon allows the dentist to practice with much greater fulfillment, confidence, and efficiency. Time is saved and the quality of treatment improves.

